# Ecological validity of don’t remember and don’t know for distinguishing accessibility- versus availability-based retrieval failures in older and younger adults: knowledge for news events

**DOI:** 10.1186/s41235-022-00458-7

**Published:** 2023-01-05

**Authors:** Sharda Umanath, Jennifer H. Coane, Mark J. Huff, Tamar Cimenian, Kai Chang

**Affiliations:** 1 Department of Psychological Science, Claremont McKenna College, 850 Columbia Ave, Claremont, CA 91711 USA; 2 Department of Psychology, Colby College, Waterville, Me USA; 3 Department of Psychology, University of Southern Mississippi, Hattiesburg, MS USA

**Keywords:** Accessibility, Availability, Retrieval failures, Phenomenology, Event memory, Aging

## Abstract

With pursuit of incremental progress and generalizability of findings in mind, we examined a possible boundary for older and younger adults’ metacognitive distinction between what is not stored in memory versus merely inaccessible with materials that are not process pure to knowledge or events: information regarding news events. Participants were asked questions about public events such as celebrity news, tragedies, and political events that were widely experienced in the previous 10–12 years, responding “I don’t know” (DK) or “I don’t remember” (DR) when retrieval failed. Memories of these events are relatively recently acquired in rich, naturalistic contexts and are likely not fully separated from episodic details. When retrieval failed, DR items were recognized with higher accuracy than DK items, both immediately and 2 years later, confirming that self-reported *not remembering* reflects failures of accessibility, whereas *not knowing* better captures a lack of availability. In fact, older adults distinguished between the causes of retrieval failures more precisely than younger adults. Together, these findings advance the reliability, validity, and generalizability of using DR and DK as a metacognitive tool to address the phenomenological experience and behavioral consequences of retrieval failures of information that contains both semantic and episodic features. Implications for metacognition in aging and related constructs like familiarity, remembering, and knowing are discussed.

**Supplementary Information:**

The online version contains supplementary material available at 10.1186/s41235-022-00458-7.

## Introduction

Successful retrieval is a fundamental expectation of a well-functioning memory. Yet, what we can access from memory fluctuates based on importance, salience, cues, frequency of previous retrieval, and context (e.g., Kornell et al., 2015; Light & Carter-Sobell, 1970; Smith & Vela, 2001; Tulving & Thomson, 1973). As such, retrieval failures are a common occurrence (Kornell & Bjork, 2009). In daily life, such experiences vary broadly, such as struggling to remember a person’s name or hesitating to fully recall a particular fact when discussing the details of the latest political exchanges. When retrieval fails, there are a range of underlying causes and associated mental experiences or phenomenological states, from the sensation of “drawing a complete blank” or having nothing come to mind, to perhaps having a tip of the tongue sensation (TOT; Schwartz, 1999). Despite the near universal nature of these experiences, it is noteworthy that much of the relevant research has focused on relatively basic retrieval from the knowledge base or on laboratory list-learning paradigms. Here, we instead explore the phenomenology associated with retrieval failures for a unique type of material: information about complex, real-world public events occurring within the previous decade or so that were, indirectly at least, “experienced” by participants in real time (i.e., as they occurred). These events are not historical, the information about them was not likely to have been learned formally, and memories for the associated details are likely linked with some episodic details in memory. We elaborate more below on the specific materials; first, we provide an overview of the relevant prior literature.

An extensive literature has investigated the TOT feeling of imminent retrieval associated with information that is just at the threshold of accessibility, including across the lifespan (Brown, 1991; Burke et al., 1991; see Schwartz, 1999; Schwartz & Metcalfe, 2014, for reviews). But of course, not all retrieval failures result in this very particular feeling (Koriat & Lieblich, 1977). As such, the literature on “feeling of knowing” (FOK), in which participants use numerical scales to rate their “feeling that one will be able to recognize—from a list of items—an item that is currently inaccessible” (Schwartz, 2006, pg. 153), has attempted to quantify the continuum of retrieval failure experiences more broadly (Hart, 1965; Koriat, 1993, 1995). Whereas TOT research typically assesses performance on vocabulary items (i.e., information stored in the knowledge base; Eysenck, 1979), FOKs have been used to examine retrieval of both general knowledge and more traditional laboratory-based episodic material (e.g., Hertzog et al., 2010; Schacter, 1983)

Building on these studies, within the context of retrieval of general knowledge, most recently, researchers leveraged natural language use, rather than numerical scales, to study the phenomenological and behavioral differences between a lack of accessibility versus availability (Coane & Umanath, 2019; cf. Tulving & Pearlstone, 1966). That is, they studied a basic difference between self-identified *not remembering* and *not knowing* and the ways in which these experiences are described in order to understand phenomenological experiences associated with retrieval failures (see also Hart, 1965; Smith & Clark, 1993). Coane and Umanath (2019) reported that participants’ definitions tended to indicate that *not remembering* reflected a temporary failure in accessibility (marginal knowledge; Berger et al., 1999), whereas *not knowing* reflected that the sought-after information was not part of the knowledge base and therefore not available. Thus, they found that these participant-generated definitions were consistent with Tulving and Pearlstone’s (1966) classic explanation of accessibility (retrievability) versus availability (storage). More specifically, from naïve participants to memory experts, participants’ definitions of Don’t Remember (DR) and Don’t Know (DK) spontaneously associated DR with a lack of access in the moment and forgetting, whereas DK was often defined as never having learned particular information at all.

The materials in Coane and Umanath’s (2019) investigation of DR/DK were from published norms of general knowledge (Tauber et al., 2013). Most of the questions from knowledge norms refer to events or information that occurred several decades prior (e.g., the name of the first cosmonaut) or are historical in nature (prior to any living age group’s lifetime) and were likely learned as part of formal education. They also refer to concepts that are relatively fixed (e.g., geography, scientific processes) or to culturally defined contexts such as literature and movies (Coane & Umanath, 2021; Nelson & Narens, 1980; Tauber et al., 2013). Such general knowledge is typically defined as “crystallized knowledge,” reflecting the long-term persistence and importantly, decontextualized nature of this information. Thus, these stimuli involve information learned long ago, in an educational context, rehearsed and retrieved often enough over time for the material to be solidified in memory, and generally not tied to specific event experiences and memories.

In fact, much of the research on the knowledge base, both in general and for older adults in particular, has examined general knowledge that is relatively stable or crystallized (Verhaeghen, 2003) and is often included in tests of intelligence or neuropsychological functioning (Kaufman & Kaufman, 1993; Wechsler, 1981). Older adults typically have strongly preserved knowledge, comparable to or exceeding that of younger adults until very late in life (see Umanath & Marsh, 2014, for a review). In sum, the materials examined in Coane and Umanath (2019) were likely devoid of episodic traces such as the time and place of acquisition, and any personally relevant details or affective responses, thus falling within the realm of semantic memory.

At the other end of the episodic/semantic memory spectrum, Lukasik et al. (2020) applied DR/DK to unanswerable questions in a traditional episodic memory context with mostly younger adults. Participants were presented with narratives and accompanying photos as the study materials. Then, they were tested on their memory for these materials in a recognition format with options to select the correct answer among lures as well as “I don’t know” and “I don’t remember” (without instructions on how or when to use these options). Critically, the test included questions regarding details that the participants had never seen, rendering those questions unanswerable; in these cases, the correct response would be “I don’t know.” Participants did respond DK significantly more often to unanswerable questions than answerable ones, providing evidence that participants were able to distinguish between using DR and DK. Lukasik et al. (2020) speculated that providing the DR response would lead to use of DK *only* when participants believed the questions were unanswerable. Instead, their collected data on participants’ explanations of how they used DR and DK generally replicated Coane and Umanath (2019), with DK being used for whenever they felt an answer was unavailable, whether because the detail was never presented or more commonly, because they thought they missed it at encoding, whereas DR was used whenever they felt the answer was available but inaccessible. Based on this work, it seems that the phenomenology associated with *not remembering* versus *not knowing* at least can be similarly experienced and effective for characterizing memories that are squarely within the realm of general knowledge and for at least one traditional episodic memory context. Further work is certainly needed in more episodic- or event memory-related studies to establish that this is wholly the case.

### Understanding retrieval failures beyond semantic and event memory

In the present work, we test the validity of DR and DK with materials that potentially exist in the gray area between episodic and/or event memory (as defined by Rubin & Umanath, 2015) and semantic memory: public news events. These events were selected to primarily include events that were somewhat “viral” in nature: very popular and receiving extensive media coverage for a few days or weeks and then being covered less frequently as new stories emerge.

Materials like these are of practical and theoretical interest for several reasons. First, they can extend traditional memory research beyond the typical single learning episode under tight experimental control and can bridge the challenge of connecting “real world” and laboratory research (Koriat & Goldsmith, 1996). This is an important step in establishing the external validity of DR and DK for capturing a lack of accessibility versus availability.

Second, such memories are typically acquired through naturalistic exposure to media (e.g., radio, television, newspapers, social media). Most laboratory studies examining long-term episodic memory include relatively simple, well-controlled stimuli and delays of less than a day (and often less than an hour), whereas laboratory studies examining semantic memory rely on vocabulary tests or general knowledge (i.e., crystallized knowledge) acquired years or decades prior. Therefore, use of such stimuli allows us to explore long-term memory processes beyond these limits (Bahrick et al., 2013) in naturalistic, non-controlled learning environments (i.e., “in the wild”). The acquisition contexts are variable in terms of modalities, source, and a host of characteristics, such as where one was when they learned of these events, whom they were with, and their emotional reactions. Clearly, these contextual elements are not typically associated with semantic memory or the knowledge base, but with episodic memory (Tulving, 1972). As is assumed by many models and theoretical approaches, however, repeated exposure to and the associated accumulation of memory traces leads to an abstraction process and the loss of episodic traces (Baddeley, 1988; Conway et al., 1997; Nelson & Shiffrin, 2013; Schank & Abelson, 1995; Versace et al., 2009).

Third, given the unique nature of these stimuli, these types of events provide an opportunity to capture information that exists in the space between the extreme ends of semantic and episodic memory: Knowledge accompanied by episodic details such as where one was when the information was learned, emotional responses, etc., but may be in the process of taking on the characteristics of semantic memory (e.g., information that is known, not remembered, decontextualized traces; Brown, 1990). Importantly, we did not assess participants’ episodic memories for these events; rather, we were interested in how they used the terms DR and DK. As reviewed above, the previous work examining use of DR and DK has been limited to materials that attempted to be purely semantic or purely episodic– something that characterizes much of memory research to date (see Rubin & Umanath, 2015, for discussion).

In recent work focused on successful retrieval, using the same materials, Coane et al. (2022) found that when younger adults and older adults retrieved fact-based details about news stories from the previous decade in an experimental task, they provided a high rate of both *remember* and *know* responses, suggesting that this information may not be fully semanticized (because *remembering* is associated with retrieval from episodic or event memory, not the knowledge base). Thus, the use of these stimuli has been previously validated, and it has been established that the populations we are examining have been exposed to the material and have preserved memory traces. Furthermore, these types of materials appear to share characteristics of both episodic and semantic memory, at least based on the phenomenological responses given by participants.

### Need for establishing the external validity of metacognitive judgments

It is also not only important, but necessary to test the effective usability of DR and DK for capturing the experiences of retrieval failures for different types of materials. Reliance on phenomenology can be problematic if participants and researchers do not consistently agree on the meaning of terms. For example, given the frequency with which older adults complain about retrieval failures (Cavanaugh et al., 1983), developing and validating ways that intuitively and consistently allow laypeople and researchers to understand the perceived causes of these failures is essential for implementing effective strategies for resolving or minimizing such challenges. Lack of clarity in how memorial experiences are described can limit the effectiveness of any intervention or limit the precision of theoretical approaches.

Bahrick et al. (2011) developed a stage model for the validation of metacognitive concepts, including naming the concept, instructions to participants, exploring the nature of participants’ phenomenological reports, and using behavioral data for validation. Coane and Umanath (2019) provided a foundation of internal validity for DR and DK. Moving beyond Bahrick and colleagues’ (2011) step of exploring participants’ phenomenological reports discussed above, younger and older adults demonstrated the metacognitive ability to use these simple phrases to effectively distinguish between a lack of accessibility versus availability when responding to general knowledge questions, behaviorally validating participants’ definitions. That is, when an initial DK response was given on a short-answer test (with or without correct answer feedback), performance on a later multiple-choice or short-answer general knowledge test was generally lower than after initial DR responses. In other words, when information was not accessible, participants were better able to recognize it among foils or recall it following feedback than when it was deemed not available.

So, here, the focus is on the next important step in validation of DR and DK to metacognitively delineate between types of retrieval failures by behaviorally testing the *external* validity of using DR and DK. Typically, external validity includes generalizability to other people, other research, and settings (Morling, 2017). Understanding the generalizability and boundary conditions for the usefulness of these terms is not only theoretically important and sound, but also necessary for effective implementation.

For comparison, consider the Remember–Know (R/K) paradigm that is used to understand the phenomenology and underlying processes of successful retrieval (Gardiner, 1988; Tulving, 1984). It also relies on participants’ understanding and correct reporting of their internal mental experiences (see Tulving, 1989, for a critique of this reliance). Despite a multitude of studies that have yielded similar findings with regards to how *remembering* and *knowing* are affected by various manipulations (see Dunn, 2004; Gardiner et al., 2002), the paradigm continues to be scrutinized for its basic face validity (Geraci et al., 2009; McCabe & Geraci, 2009; Perfect et al., 1996; Rubin & Umanath, 2015; Strack & Forster, 1995; Williams & Moulin, 2015; Yonelinas, 2002; for a review, see Umanath & Coane, 2020). That is, participants require extensive instructions (Barber et al., 2008; Gardiner & Java, 1990; Rajaram, 1993; Yonelinas, 2002) and a very particular experimental context (e.g., Gardiner, 1988) for the terms to successfully map onto recollection and familiarity—which is what the vast majority of researchers up to this point have been using the terms to understand (see Umanath & Coane, 2020, for a review). Even so, slight modifications in the instructions lead to large differences in usage and performance (Eldridge et al., 2002; Geraci & McCabe, 2006; Geraci et al., 2009; McCabe et al., 2011; Rotello et al., 2005; Williams & Lindsay, 2019).

The current work explicitly attempts to prevent such a disconnect between participants and researchers in using DR and DK for capturing and characterizing experiences of retrieval failure from the outset, rather than discovering such a fundamental issue after extensive (potentially problematic) usage. We examine the extent to which the terms DR and DK effectively distinguish accessibility versus availability failures for other settings in the form of a different set of materials described below by using complex, naturalistically acquired common events occurring over the previous decade and continues to consider generalizability to other people with samples of older adults. If the original findings turn out to be constrained to a specific type of knowledge, clearly, the use of DR and DK will be limited in its scope and application.

### The present research

Two waves of data were collected to examine the generalizability of the phenomenology of retrieval failures for real-world knowledge for events from public news media. Our stimuli were brief descriptions of relatively recent (2006–2016) news stories regarding a variety of topics from politics to pop culture and natural disasters.

Under the circumstances specific to such stimuli, do DR and DK mean the same things and are they used in the same ways as prior work has found? This is the empirical question we aim to answer. In particular, for materials that are potentially familiar—due to their viral nature—but not accessible in the moment, use of DK might take on more of a face-saving role: Rather than admitting a failure in remembering a detail from a public news event, participants might prefer to use DK to signal a lack of certainty or an unwillingness to commit to an answer (Smith & Clark, 1993).

Samples of older adults participated in the present studies to consistently address generalizability of these metacognitive measures across age. Older and younger adults differ along a number of dimensions, especially those of relevance to the present questions, as mentioned above: memory, knowledge, and metacognition. Older adults tend to attend to news more than younger adults, at least traditional news media like radio, newspapers, and television (Bourne et al., 2020). As such, older adults would, overall, outperform younger adults in overall accuracy and might experience more DR responses, indicating an awareness that the information is available, albeit temporarily inaccessible, consistent with greater and maintained general knowledge (Park, 2000) as well as overall increased experience of retrieval failures (Cavanaugh et al., 1983). However, older adults also tend to perform worse than younger adults on traditional episodic tasks and have well-documented deficits in episodic metacognition (e.g., Souchay et al., 2000, 2007; Thomas et al., 2011), encoding new information (Balota et al., 2000; Park, 2000), and even report this themselves (Hertzog & Dixon, 1994). However, in semantic tasks, older adults’ self-assessments are as accurate as those of younger adults (e.g., Backman & Karlsson, 1985; Hertzog & Dunlosky, 2011; Lachman et al., 1979; Morson et al., 2015). Thus, more DK responses might occur, reflective of an absence of information stored in memory, if the information was simply not encoded or had decayed. Older adults might also use DK more often to reflect uncertainty or to save face: In this case, accuracy for DK items would reflect an underestimation of knowledge (Smith & Clark, 1993; see Coane & Umanath, 2019).

Study 2 was a replication of Study 1 in which we extended the retention interval of these naturalistic stimuli by approximately 18 months. Given the novelty of the stimuli and the relative paucity of work demonstrating how usage of DR and DK map onto memory performance and metamemory accuracy, and in the spirit of reproducible science, replications help establish the reliability of an effect. Hereafter, we refer to the two testing points as Wave 1 and Wave 2, to emphasize the similarity across them and the fact that this was not a longitudinal study examining forgetting at the individual level. The second wave of testing did allow us to extend the retention interval for the events. In particular for the younger adults tested in Wave 2, some of the events occurred in early elementary school. Therefore, the difference between *not remembering* and *not knowing* might have been less salient, because the familiarity of those events decreased, thereby compressing the range of stored information toward lower levels of retention.

In addition to assessing objective memory performance for these items, we also obtained a measure of self-rated familiarity for each event. Importantly, these ratings were collected prior to participants answering specific questions. Thus, familiarity was evaluated prior to any explicit retrieval attempt (although it is likely that some covert or implicit retrieval took place in assessing the event’s familiarity). Therefore, we could assess the familiarity of items subsequently given a DR or DK response when an explicit retrieval attempt failed. Coane et al. (2022) found that retrieval success of these public events was associated with both the phenomenological indicators of *knowing* (retrieval from semantic stores) and *remembering* (retrieval from episodic stores). Furthermore, *know* responses were more accurate than *remember* responses in a subsequent multiple-choice task, whereas familiarity, perhaps surprisingly, did not differ as a function of phenomenological responses. Here, we mirrored this work, but focused on retrieval failures. Given prior evidence that DR responses are associated with inaccessible information and DK responses with unavailable information, familiarity should be higher for items subsequently given a DR response than those given a DK response. Furthermore, if DR responses are given when retrieval failure is only temporary, familiarity of DR items might be similar to that of items correctly answered. Alternatively, early assessments of familiarity might predict subsequent retrieval failures, such that DR responses are associated with lower familiarity than correct responses indicating relative accuracy in how participants assess the ease of retrieval.

The work described below is meant to provide an incremental contribution and further assurance of the replicability and validity of the use of DR and DK. In light of the replication crisis currently affecting the field of psychology (and other disciplines; Nosek & Errington, 2020; Nosek et al., 2022), it is crucially important to demonstrate that novel findings are, indeed, robust to replication across different factors. As argued by Nosek and Errington (2020), “The purpose of replication is to advance theory by confronting existing understanding with new evidence” (p. 3). Thus, as mentioned above, here we provide new evidence to critically evaluate the extent to which our earlier claim—that *not remembering* and *not knowing* map onto retrieval failures of accessibility and availability, respectively—is robust across variations in participants, stimuli, and historical context. Thus, our contribution with the present work is to provide an examination and potential validation of older and younger adults’ accurate metacognitive usage of DR and DK for information about public news media, content that differs from the previously explored materials in a myriad of ways described above. This represents an important step in not only establishing the external validity of DR and DK, but also in furthering our understanding of older adults’ metacognition.

## Method

### Participants

Participants in Wave 1 were 32 older adults (OAs) drawn from the greater Kennebec County community (72% female), and 33 undergraduate students from Colby College (73% female). Participants in Wave 2 were 42 OAs (67% female), and 41 undergraduate students (78% female) from the same populations but who had not participated in the earlier study. That is, prospective participants were excluded from the possibility of participating in Wave 2 if they had participated in Wave 1. All participants were tested in the lab and were compensated at a rate of $10 per hour. Younger adults (YAs) were also given the option of earning course credit for an introductory psychology course, in lieu of monetary compensation. Sample sizes were determined based on those in Coane and Umanath (2019) to ensure equivalent power. All participants completed the Shipley (1940) Vocabulary scale, in order to assess general cognitive ability. In Wave 2, OAs were also administered the Mini-Mental State Examination (MMSE; Folstein et al., 1975). See Table 1 for demographic information. Data collection for Wave 1 took place between February and March of 2017 and for Wave 2 between June and October of 2018.

**Table 1:** Demographic information for all participants (standard deviations and range in parentheses)

	Wave 1		Wave 2	
	Younger adults	Older adults	Younger adults	Older adults
Age	18.94 (1.06; 18–22)	72.19 (5.37; 63–85)	19.24 (1.11; 18–22)	71.27 (6.64; 62–97)
Shipley vocabulary	32.75 (3.08; 25–38)	35.63 (3.64; 27–40)	28.98 (3.84; 22–35)	35.02 (4.12; 22–39)
MMSE	–	–	–	29.40 (1.04; 26–30)

### Materials and procedure

The stimuli consisted of news events from the years 2006–2016. Events were initially selected by consulting a variety of internet sites for the “Top 10 News Stories” of each year and covered a variety of topics, including political events, national and international tragedies, and pop culture. Using Google Trends, we verified that the events had a clear peak in popularity in terms of internet searches. For each potential event, we developed a one-sentence description (e.g., death of Eric Garner) for an initial familiarity rating task and a question about a specific detail about that event (e.g., How did Eric Garner die?). Pilot testing was conducted online in the summer of 2016, in which separate groups of participants recruited on Amazon’s Mechanical Turk platform rated each item’s familiarity (on a 5-point scale) or answered a specific question about the event. The familiarity task was completed by 81 participants (24 women, 1 other; *M*_*age*_ = 33.78, *SD* = 10.88; range = 20–64) and the question task was completed by 81 different participants (35 women; *M*_*age*_ = 32.08, *SD* = 8.08; range = 19–57). Items were presented in a different random order for each participant. An 87-item subset of the initial 178 stimuli were chosen, selected to be moderately familiar (*M* = 4.49, *SD* = 1.0) and to have a range of difficulty levels (*M* = .48, *SD* = .21, range 0–1). All participants responded to all 87 stimuli, and the probe for the initial familiarity rating did not include the answer to the question probe. Further details on the stimulus development and selection can be found in Coane et al. (2022). In Wave 2, 13 additional items were added that referred to events from 2016 to 2018; analyses on those items are not discussed here. In addition, for Wave 2, some of the stimuli required minor modifications (e.g., one of the original questions was about the name of Prince William and Kate Middleton’s child; however, when data were collected in Wave 2, they had more than one child). We note that participants were not asked about their personal recollections or reactions to the events but only about the factual nature of them (in other words, we were interested in their objective memory for the events). The familiarity rating task provided a subjective assessment of the contents of their memory.

The study was programmed using E-Prime 2 software (Schneider et al., 2012). Participants were tested individually or in small groups (OAs were only tested individually). Each participant gave informed consent and completed a demographics information form prior to completion of the online survey. The study consisted of three phases: familiarity, short-answer questions, and multiple-choice recognition. In the familiarity task, participants were presented with a general description of each event in randomized order. For each event, such as “Saddam Hussein’s death,” participants were asked to rate their familiarity with the event on a scale of 1 (low familiarity) to 5 (high familiarity).

In the short-answer phase, participants were asked questions about a specific detail for each of the 87 events (e.g., “How was Saddam Hussein executed?”) and prompted to type a response. Participants were told they could use “I don’t know” (DK) and “I don’t remember” (DR) if they could not provide an answer to a question. As in Coane and Umanath (2019), no instructions were provided regarding when or how to use these options. Following the completion of the short-answer phase, participants completed a 5-min filler task (either a Sudoku puzzle or a set of complex multiplication problems).

In the final phase, the multiple-choice recognition task, participants were given the same questions, in a new randomized order, in a multiple-choice format with the correct answer and three foils. For example, the question asking how Eric Garner died included, in addition to the actual cause of death (*choking* or *asphyxiation*), the alternatives *shot*, *tasered*, or *run over*. Each response option was numbered (1–4), and participants made their responses using the computer’s external keyboard. The correct response occurred approximately an equal number of times in each position. Items were randomized anew for each participant in all three phases.

At the end of the study, participants were asked to indicate how they had used DR and DK throughout the study by answering the questions “What did you mean when you used ‘I don’t remember/know’ in the first part of the study?”.

## Results

For all results, we included a Bayesian hypothesis test to supplement the analyses conducted using null-hypothesis-significance testing. The Bayesian test computes a Bayes factor (BF), which reflects a numerical value that quantifies the predictive capacity of the null hypothesis model (H0) compared to the alternative hypothesis model (H1). For reported BFs, the subscript reported corresponds to hypothesis that the BF favors, either H1 over H0 (BF_10_) or H0 or H1 (BF_01_). Although several interpretive criteria have been proposed, we describe the values reported by Doorne et al. (2021). For null hypothesis evidence, BF_10_s less than 1/10 suggest strong evidence for the null, BF_10_s between 1/10 and 1/3 indicate moderate evidence for the null, and BF_10_s between 1/3 and 1 indicate weak evidence for the null. For alternative hypothesis evidence, BF_10_s greater than 10 indicate strong evidence for the alternative, BF_10_s between 3 and 10 indicate moderate evidence for the alternative, and BF_10_s between 1 and 3 indicate weak evidence for the alternative. Despite these interpretive criteria, we caution against applying these values as all-or-none cutoffs for making data conclusions and not to conflate BFs as general estimates of effect size (Additional file 1).

Short-answer responses were coded as incorrect (including errors and no answer given), correct (including minor spelling errors or morphological variations), DR, or DK. In all analyses reported below, we include correct, DR, DK, and incorrect responses (see Table 2). Most incorrect responses were errors of commission, as only 42 responses [.4%] were blank. Familiarity and objective accuracy data were submitted to 2 (Age) × 4 (Response) × 2 (Wave) mixed ANOVAs, where Age and Wave were between-subjects factors.^1^ We included Wave as a factor to better highlight any changes (or lack thereof) in response distributions and patterns across the 18 month delay.

**Table 2:** Proportion of responses during the short-answer task in YAs and OAs in Waves 1 and 2 (standard error of the mean in parentheses)

	Wave 1				Wave 2			
	Correct	DR	DK	Commission error	Correct	DR	DK	Commission error
YAs	.40 (.03)	.13 (.02)	.39 (.03)	.08 (.01)	.27 (.03)	.17 (.02)	.46 (.03)	.09 (.01)
OAs	.28 (.02)	.21 (.02)	.33 (.02)	.17 (.02)	.29 (.02)	.23 (.02)	.31 (.02)	.16 (.01)
*p*-value	.005	.002	.111	< .001	.625	.010	< .001	< .001

### Initial familiarity ratings

**Fig. 1:**
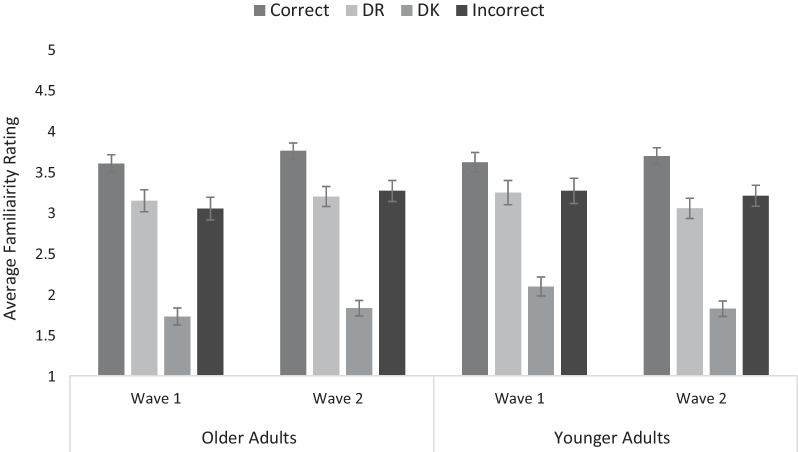
Average familiarity as a function of age, wave, and response type (error bars represent standard error of the mean)

The familiarity analyses included data from 32 OAs in Wave 1, 38 OAs in Wave 2, 26 YAs in Wave 1, and 37 YAs in Wave 2 (some participants did not use all response options; see Fig. 1). Items correctly answered during encoding were given the highest ratings (*M* = 3.66, SEM = .05), followed by those given a DR response (*M* = 3.16, SEM = .07) or incorrect response (*M* = 3.19, SEM = .07), with those given a DK response being rated least familiar (*M* = 1.84, SEM = .05), *F*(2.72, 35.92) = 448.75, *p* < .001, *η*_*p*_^2^ = .78, BF_10_ = 2.659 × 10^13^. All pairwise comparisons were significant (all *p*s ≤ .001) except for DR and incorrect responses, which did not differ from one another (*p* ≥ .999). No other effects were significant, all *F*s ≤ 1.98, *p*s ≥ .122, BF_10_s < .121. Thus, subjective familiarity appeared to remain stable across time points and as a function of age.

### Initial short-answer task

Turning to performance during the short-answer task, we examined responses as a function of Age and Wave. Given the lack of independence in the response options (e.g., if Correct responses increase, changes in the other three categories necessarily have to decrease), we report separate analyses for each response option. Doing so also provides more insights into any differences due age or time. For all analyses, 32 OAs’ and 33 YAs’ data were included from Wave 1 and 42 OAs’ and 41 YAs’ data were included from Wave 2.

The proportion of correct responses declined from Wave 1 to Wave 2, *F*(1, 144) = 6.10, *p* = .015, *η*_*p*_^2^ = .04, BF_10_ = 4.512, from 0.34 (SEM = .02) in Wave 1 to .28 (SEM = .02) in Wave 2. The effect of Age approached significance, *F*(1, 144) = 3.85, *p* = .052, *η*_*p*_^2^ = .03, BF_10_ = 1.610, with YAs (*M* = .33, SEM = .02) correctly answering more questions than OAs (*M* = .28, SEM = .02). These effects were qualified by a significant interaction, *F*(1, 144) = 6.71, *p* = .011, *η*_*p*_^2^ = .05, BF_10_ = 4.632, which reflected the fact that OAs’ accuracy did not change from Wave 1 to Wave 2 (*M* = .28 and *M* = .29, respectively), *F* < 1.0, *p* = .932, BF_10_ = .243, whereas YAs answered more questions correctly in Wave 1 (*M* = .40, SEM = .03) than in Wave 2 (*M* = .27), *F*(1, 144) = 12.84, *p* < .001, *η*_*p*_^2^ = .08, BF_10_ = 2.373 × 10^7^.

In the analysis on DR responses, only the effect of age was reliable: OAs (*M* = .22, SEM = .01) gave a DR response more frequently than YAs (*M* = .15, SEM = .01), *F*(1, 144) = 17.10, *p* < .001, *η*_*p*_^2^ = .11, BF_10_ = 263.841. The effect of Wave was not significant *F* = 2.26, *p* = .135, BF_10_ = .554, nor was the interaction, *F* < 1.0, *p* = .486, BF_10_ = 0.298.

Similarly, the proportion of DK responses only differed as a function of Age, *F*(3, 144) = 15.19, *p* < .001, *η*_*p*_^2^ = .10, BF_10_ = 229.832, with YAs (*M* = .43, SEM = .02) giving it as a response more than OAs (*M* = .32, SEM = .02). Neither the effect of Wave nor the interaction was significant, both *F*s ≤ 2.63, *p*s ≥ .107, BF_10_s < 0.709.

Incorrect responses also differed by age, *F*(1, 144) = 41.63, *p* < .001, *η*_*p*_^2^ = .22, BF_10_ = 3.174 × 10^6^. OAs (*M* = .16, SEM = .01) were more likely to provide an incorrect response than YAs (*M* = .09, SEM = .01). In sum, examination of the relative proportion of responses indicates that correct responses declined over time, but only for YAs, whereas DR, DK, and incorrect responses were relatively stable but differentially frequent in YAs and OAs.

### Final multiple-choice recognition task

**Fig. 2:**
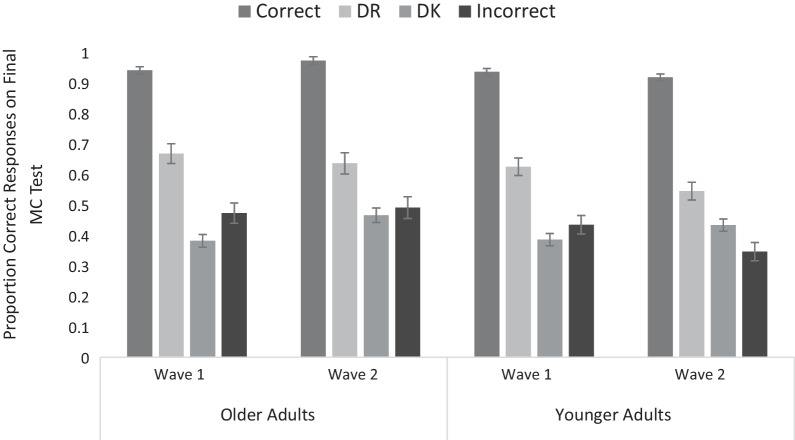
Accuracy on the final MC test in Waves 1 and 2 as a function of response on the initial test and age (error bars reflect standard error of the mean)

We examined the proportion of correct responses as a function of the response given during the short-answer task, with Age and Wave as between-subjects factors. This analysis included data from 63 YAs (26 in Wave 1 and 37 in Wave 2) and 70 OAs (32 and 38 in Waves 1 and 2, respectively) because some participants were missing data in some of the cells. Means are presented in Fig. 2.

The overall proportion of correct responses on the MC test declined from Wave 1 (*M* = .63, SEM = .012) to Wave 2 (*M* = .58, SEM = .01), *F*(1, 129) = 10.42, *p* = .002, *η*_*p*_^2^ = .075, BF_10_ = 7.350. The main effect of age was not significant, *F* < 1.0, *p* = .773, BF_10_ = 0.411. Overall, correct responses for items initially correctly recalled in the short-answer task (*M* = .94, SEM = .01) were almost at ceiling, followed by correct responses to items given a DR response (*M* = .62, SEM = .02), with lowest accuracy for items given a DK response (*M* = .42, SEM = .01) and for those given an incorrect response (M = .43, *SEM* = .02), *F*(2.48, 319.70) = 432.06, *p* < .001, *η*_*p*_^2^ = .77, BF_10_ = 3.689 × 10^13^. All pairwise comparisons were significant (all *p*s < .001, BF_10_s > 9.590 × 10^12^), other than DK vs. incorrect responses (*p* > .999, BF_10_ = 0.159).

Age and Response interacted, *F*(3, 387) = 5.32, *p* = .001, *η*_*p*_^2^ = .04, BF_10_ = 16.486. Whereas accuracy between YAs and OAs was the same for initially correct items *F*(1, 133) < 1.0, *p* = .494, BF_10_ = 0.192, OAs were slightly, albeit not significantly, more accurate than YAs for initially not remembered items *F*(1, 135) = 3.684, *p* = .057, *η*_*p*_^2^ = .02, BF_10_ = 0.973, whereas the opposite was true for initially not known items *F*(1, 129) = 9.393, *p* = .003, *η*_*p*_^2^ = .06, BF_10_ = 12.125. Final accuracy on initially incorrect items did not differ across age groups, F(1, 142) = 1.261, p = .263, BF_10_ = 0.381. Thus, OAs were more accurate than YAs at distinguishing between not remembered and not known items. None of the other effects or interactions were significant, all *F*s ≤ 3.63, *p*s ≥ .059, BF_10_s > 1.507.

Interestingly, this suggests that, for this particular type of stimulus, YAs are worse than OAs at recognizing what they do not know or do not remember. OAs showed greater sensitivity than YAs to perceived differences in retrievability based on initial assessments of *not remembering* and *not knowing*. The critical finding—that final MC test performance was better for items that had been identified as *not remembered* than those identified as *not known* (all pairwise comparisons were significant at *p* < .001, BF_10_s > 19,440.263)—does support the hypothesis that *not remembering* is based on different phenomenological cues than *not knowing* and that participants can make this distinction, regardless of age. Interestingly, although initial familiarity for incorrect responses was similar to that for not remembered items, in terms of actual objective accuracy on the final test, both YAs’ and OAs’ performance was more similar to that of not known items. This suggests that familiarity is not as reliable a source of information when distinguishing between accessibility- and availability-driven retrieval failures.

### Participant definitions of DR and DK

For both waves, all valid responses for defining what DR and DK meant were scored across the dimensions previously found to be most associated with *not remembering* and *not knowing* in Coane and Umanath (2019). Coding and analyzing these definitions allowed us to attempt to continue to replicate and extend participants’ definitions from online participants (Coane & Umanath, 2019) to participants in the lab environment, to OAs, and to situations in which the definitions were provided in the context of prior retrieval attempts of information that includes episodic and semantic properties.

To preview, participants’ definitions generally replicated the prior work. Importantly, here, participants in these studies were asked to define what they meant by DR and DK at the end of the studies, *after* they used the terms. Rather than defining the terms without any context whatsoever, these participants had already made use of the terms, without any explicit definitions or instructions from the experimenters. Such experience with the stimuli could have influenced participants’ definitions, priming them to define the terms in the context of the tasks they just completed. Indeed, Coane and Umanath (2019) found that the distinctions in participants’ definitions of DR and DK were even more stark after they had attempted to answer general knowledge questions compared to when lay people were simply asked to define the terms in isolation. Similarly, Lukasik et al. (2020) demonstrated shifted definitions based on their episodic memory paradigm that included unanswerable questions.

The present analyses focused on the four constructs that emerged as most important in prior work: Accessibility, Never, Forgetting, and Availability. A response was considered to use Accessibility when it included the inability to retrieve information particularly in the moment, though it was likely stored. Never was coded as present whenever a response explicitly included the word “never,” typically in the context of never having been learned or encountered. A response was coded as including Forgetting when a participant made explicit reference to the loss of information over time. Availability was coded as present whenever a response referred to storage—that the information is or was stored in memory on the one hand or never stored at all, on the other hand. For further details of these dimensions, the general coding schemes, and examples, please see Coane and Umanath (2019). For each dimension, a score of 1 meant that the dimension was referenced in the participant definition and a 0 indicated that it was absent. Note that a single response could be coded as referencing multiple dimensions. Responses were coded for all dimensions by two independent coders and correlations between the coders ranged from .91 to .99 for Waves 1 and 2. Discrepancies were resolved through discussion. Responses that simply restated the terms (e.g., “I used IDK when I didn’t know”) or were not relevant to the question were not analyzed.

A 2 (Wave: 1, 2) × 2 (Age: YA, OA) × 2 (Question: DR, DK) MANOVA was also conducted on the four constructs. For all marginal means, see Table 3. This analysis yielded a significant effect of Question, Hotelling’s Trace = 9.76, *F*(4, 124) = 302.52, *p* < .001, *η*_*p*_^2^ = .91, a main effect of Age, Hotelling’s Trace = .09, *F*(4, 124) = 2.68, *p* = .04, *η*_*p*_^2^ = .08, and a Question × Age interaction, Hotelling’s Trace = .09, *F*(4, 124) = 2.69, *p* < .03, *η*_*p*_^2^ = .08. No effects including Wave were significant, *p*s > .33. Therefore, we proceed with presenting and interpreting each univariate ANOVA.

**Table 3:** Proportion of participants’ responses referencing dimensions as a function of question, age, study, and dimension in Waves 1 and 2 (standard error of the mean in parentheses)

	Wave 1				Wave 2			
	OA		YA		OA		YA	
	DR	DK	DR	DK	DR	DK	DR	DK
Accessibility	.65 (.09)	.04 (.04)	.79 (.08)	.03 (.03)	.57 (.07)	.03 (.03)	.88 (.07)	.03 (.03)
Never	.00 (.00)	.87 (.07)	.00 (.00)	.91 (.06)	.00 (.00)	.80 (.07)	.00 (.00)	.93 (.05)
Forgetting	.52 (.11)	.00 (.03)	.49 (.09)	.00 (.02)	.46 (.09)	.00 (.02)	.50 (.08)	.05 (.02)
Availability	.96 (.03)	.96 (.06)	1.00 (.02)	.97 (.05)	.97 (.02)	.83 (.05)	1.00 (.02)	.93 (.04)

There were significant main effects of Question for all four constructs. For Accessibility, participants referred to this construct significantly more often for DR (*M* = .72, *SEM* = .04), than for DK [*M* = .03, *SEM* = .02; *F*(1, 127) = 299.78, *MSE* = .10, *p* < .001, *η*_*p*_^2^ = .70). In addition, YAs referenced it more often than OAs, *F*(1, 127) = 5.98 *MSE* = 18, *p* = .02, *η*_*p*_^2^ = .05. This was qualified by an Age x Question interaction [*F*(1, 127) = 8.19, *MSE* = .10, *p* = .005, *η*_*p*_^2^ = .06] such that for DK, there was no difference in references (*M*s = .03 vs .04, *SEM*s = .02), but for DR, YAs (*M* = .83, *SEM* = .05) were more likely to reference Accessibility than OAs (*M* = .61, *SEM* = .06). This pattern provides a hint that when to-be-retrieved items have some episodic qualities, such as memories for the acquisition context or one’s emotional responses, difficulties with access are even more pronounced, especially for YAs who may be attempting to retrieve more details than OAs. Additionally, it could be the case that OAs semanticize information more quickly, retaining fewer episodic details. OAs typically do show more reliance on gist-based processing and increased deficits in source-based recollection (Balota et al., 2000) and, thus, might rely more heavily on prior knowledge (Umanath & Marsh, 2014) and their retrieval might be lacking those specific details, whereas the general elements of an event might still be available.

For Forgetting, there were no significant effects (*p*s > .50) other than that of Question, so regardless of Age or Wave, participants referred to this construct almost exclusively for DR (*M* = .49, *SEM* = .05) versus for DK [*M* = .01, *SEM* = .01; *F*(1, 127) = 112.14, *MSE* = .13, *p* < .001, *η*_*p*_^2^ = .47]. Again, in contrast, participants only referred to Never for DK (*M* = .88, *SEM* = .03) compared to DR [*M* = .00, *SEM* = .00; *F*(1, 127) = 892.83, *MSE* = .05, *p* < .001, *η*_*p*_^2^ = .88], but no other effects reached significance (*p*s > .16). For Availability, similar to prior work, participants were essentially at ceiling in referencing this dimension. However, there was a statistically significant difference such that participants referenced this dimension more for DR (*M* = .98, *SEM* = .01) than for DK [*M* = .92, *SEM* = .03, *F*(1, 127) = 6.12, *MSE* = .04, *p* = .02, *η*_*p*_^2^ = .05]. In such a situation, interpreting this difference as genuinely meaningful seems inappropriate.

In sum, for all constructs, the overall effects replicated Coane and Umanath (2019), with Accessibility and Forgetting being referenced much more for DR than DK, Never being used almost exclusively for DK, and Availability essentially being referenced across the board, likely because storage is relevant in considering both terms. These data further validate and extend the effective use of DR and DK in that 1) the terms are considered similarly for materials that are acquired and rehearsed “in the wild” via various uncontrolled means, are not solely knowledge or events as typically used in laboratory studies, and after three previous exposures to the stimuli in question (i.e., the familiarity task and both question phases). Thus, this distinction is robust even after multiple retrieval attempts, and 2) for such stimuli, OAs show the same patterns of defining the terms as YAs, though they may reference some of them with less frequency than YAs.

## General discussion

Memory for news events varies in both availability and accessibility, and both YA and OA participants make this distinction in both subjective familiarity and objective accuracy measures. When information is deemed *not known*, it is rated as less familiar and subsequent recognition fails often. Conversely, information that is *not remembered* is rated as more familiar and is more likely to be correctly recognized. Thus, the phenomenology associated with retrieval failures due to different causes manifests itself behaviorally. Additionally, the fact that items given a DR response were rated lower in familiarity and then resulted in lower performance on the final test than items correctly answered in the initial short-answer phase suggests that information that is stored but cannot be retrieved at a specific point in time is in fact less accessible or less “complete” than information readily accessible. Interestingly, items given a DR response and incorrectly answered items were rated as equally familiar, even if final test accuracy differed, suggesting there are different bases for assessing familiarity and for actual retrieval and that familiarity may not be as predictive a cue. It is worth noting that, overall, the pattern of results showed remarkable stability across the two waves of data collection. As might be expected, younger adults were less able to retrieve the correct answer for more events in Wave 2 than in Wave 1, but no differences emerged for familiarity or final test accuracy, and this pattern was not driven by the oldest events. We discuss this more fully below. These data are consistent with Coane and Umanath (2019) and extend the pattern to memory for information about news events that are acquired and rehearsed “in the wild” via various uncontrolled means, are not solely knowledge or events as typically used in laboratory studies, and likely include both episodic characteristics and semantic properties (Brown, 1990; Coane et al., 2022).

It is worth noting that such a conclusion was not foregone. For example, DK can be used in natural language use as reflecting uncertainty, as in being unsure if a stimulus was presented or being unsure if someone actually encoded a stimulus. Research in pragmatics suggests that use of DK in natural language can reflect, as we assume here, a lack of knowledge. However, it can also be used as a face-saving tool by avoiding commitment to an answer, general uncertainty, a desire to withhold information, or an attempt to soften a disagreement (Tsui, 1991). Given the breadth of uses of this common expression in everyday language, our aim was to continue to build the foundation of general usability of DR and DK for understanding different kinds of retrieval failures, particularly beyond materials that are attempting to tap and assess the contents of one memory store versus another. Specifically, we needed to establish that, across materials, populations, and settings, DK does consistently reflect lack of knowledge.

This is a critical step forward in establishing the external validity of the participant use of DR and DK which refer to phenomenological experiences to successfully capture and distinguish between the underlying causes of retrieval failures as a lack of accessibility versus availability. Furthermore, they demonstrate the replicability of the earlier findings (Coane & Umanath, 2019), which is fundamentally important when considering the ongoing challenges undermining trust in science in general (Edlund et al., 2022). First, both older adults and younger adults continued to demonstrate the ability to distinguish between retrieval failures due to a lack of accessibility versus availability (see also, Lukasik et al., 2020). Second, this pattern persisted even with the present materials (see also, Coane et al., 2022). That is, both age groups were much less likely to recover answers to questions for which they had initially thought they did not know compared to those they claimed they did not remember. Third, indeed, participants’ definitions of DR and DK also remained consistent for these novel materials. Fourth, it is worth highlighting that even after multiple retrieval attempts, DK did not appear to be associated with a lack of certainty (as evidenced by the high rate of Never references).

### Maintenance of retrieval failure-related metacognition in aging

For the present materials, older adults showed the ability to differentiate between what was unavailable and what was inaccessible. Remarkably, older adults were even better at this separation than their younger counterparts. Specifically, they correctly answered *more* items originally classified as not remembered and *fewer* items classified as not known relative to younger adults. Thus, excitingly, older adults metacognition regarding their retrieval failures seems to be rather robust. This stands in contrast to some of the concerns raised about the RK paradigm with particular populations, such as older adults. For example, Aggleton et al. (2005) noted that individuals with amnesia might fail to retain the instructions for how to use the terms over an extended period of time. Bowler et al. (2000) and Williams and Moulin (2015) raised similar concerns for individuals with other forms of cognitive decline, and McCabe and Geraci (2009) noted that even healthy older adults might experience some difficulty, in part because their additional linguistic experience might result in more fixed interpretations of the terms *remember* and *know*. Some of these issues may arise because indeed, in the natural language use of lay people, non-memory psychology experts, and even memory experts, *remember* actually maps onto retrieval of events and *know* maps roughly onto retrieval from the knowledge base, among other things, in general accordance with Tulving’s original conception (Tulving, 1985; Umanath & Coane, 2020).

Coane and Umanath (2019) found that older adults tended to underestimate their knowledge when answering general knowledge questions, performing well above chance on items initially classified as *not known*. It is worth noting that the greater recovery of knowledge in older adults relative to younger adults did not appear to emerge for the present materials. Here, although both younger and older adults performed above chance on items given a DK response (all *p*s < .001, where pure chance would be considered set at .25), younger adults tended to underestimate their ability to access information more than older adults. Younger adults also gave slightly higher familiarity ratings to *not known* items, suggesting they might have had greater familiarity with the general topic or been able to access related knowledge (cf. Koriat, 1993). Interestingly, given that greater familiarity is associated with items that are *not remembered* than those that are *not known*, the sense of familiarity might be one of the phenomenological dimensions used by participants in determining whether information is indeed stored in memory or not.

Additionally, an important factor to address here is what “chance” really means in this context. For the final 4-alternative multiple-choice recognition test, answering correctly due to random chance is .25. However, this assumes guessing without any related knowledge or ability to rule out alternatives. The more an individual knows, which is typically more in the case of older adults versus younger adults, the “correct due to chance” level rises as they are able to rule out lures. One could argue that responding DR or DK and then selecting the correct answer with a probability of 50% on the final MC test might reflect accurate metacognition, in that one could combine guessing with an exclusion of incorrect options.

If nothing else, these results underscore the importance of using a variety of materials in terms of both difficulty and richness to develop a more complete and accurate understanding of the complex interactions between memory, metacognition, and age. As discussed in Introduction, older adults often perform poorly on episodic tasks, both in terms of memory accuracy and metacognitive calibration. With the present stimuli, it was possible that the episodic-like characteristics of the stimuli could have negatively impacted older adults’ performance. However, this was clearly not the case. Furthermore, our findings suggest that information that is not canonically part of the knowledge base still can be readily distinguished in terms of phenomenology associated with retrieval failures.

### Further support for a DR/DK distinction

As mentioned above, participants’ definitions of DR and DK were remarkably consistent despite different sets of participants using the terms for different kinds of materials. There was little support for age differences regarding the way in which participants defined what it means to say “I don’t know” versus “I don’t remember.” Though they showed the same basic patterns as younger adults, older adults sometimes referenced particular dimensions less often. The difference was in references to (a lack of) accessibility for DR. Regarding the decreased references to accessibility for DR in Waves 1 and 2, one possibility is that this is an artifact of the materials. A number of stimuli used were from the realm of pop culture or celebrity news, which older adults might not attend to as much. From a value-directed memory perspective (Castel, 2007; Castel et al., 2011), this lack of interest might, in turn, affect how they overall defined the terms. Specifically, it is possible that, after being asked a number of questions about topics outside of their interest, they considered accessibility slightly less and focused more on the lack of availability (i.e., this information is likely not in my memory because I am not interested in it).

Interestingly, familiarity ratings further discriminated between DR and DK responses. Participants rated *not remembered* items as more familiar than those that were *not known*. This pattern supports the notion that familiarity is an underlying phenomenological cue used by participants in deciding whether or not information is stored in memory, consistent with work on FOKs and judgments of learning (Hart, 1965; Metcalfe et al., 1993; Schwartz, 2006). Indeed, in the context of an episodic memory task that included unanswerable questions, Lukasik et al. (2020) reported that many participants’ explanations of their use of DR “mentioned that the queried detail seemed familiar but there was not enough specific information available in order to answer the question” (p. 1304). Furthermore, in participants’ definitions of DR and DK, the word “never” is much more often used with DK, across materials, indicating no familiarity whatsoever. Notably, once public event stimuli cross the threshold into accessibility and successful retrieval, familiarity is no longer a distinguishing quality between *remembering* and *knowing.* In previous work, Coane et al. (2022) found that familiarity ratings for public events were equivalent between accurate *remember* and accurate *know* responses. This suggests that the role of familiarity might be more dominant when retrieval is more difficult or simply at the earlier stages of assessment/metacognitive evaluation, consistent with the accessibility or heuristic model of FOK judgments (Koriat, 1993, 2000) and cue familiarity account (Metcalfe et al., 1993). In the present work, although initial familiarity for incorrect and DR responses was the same, actual performance was not. Furthermore, younger and older adults gave similar familiarity ratings, even though objective accuracy differed. This suggests that not remembering seems to involve a qualitatively different process than assessing familiarity—something can “feel familiar” but still be below the threshold of a failure in accessibility. One possibility is that familiarity might be capturing something along the lines of “I used to know this.” Clearly, however, reliance on familiarity appears to be less fine-grained than reliance on the phenomenologies associated with more specific causes of retrieval failures. In concert with Coane et al. (2022), who reported higher levels of familiarity for the present stimuli when participants claimed to *know* the correct answer rather than *remember* it, it appears that familiarity is distinct from both *knowing *and *not knowing* and relies on potentially different information or processes. Clearly, given the many ways in which familiarity is used in memory research (e.g., Yonelinas, 2002; see Umanath & Coane, 2020, for a recent review) further exploring the ways in which researchers and lay participants conceptualize and use the term is worthy of future work.

### Insights into the episodic/semantic distinction

Our results can provide insight into the critical theoretical distinction between episodic or event memory and semantic memory (see special issue in *Memory & Cognition*). The public news events examined here possess both episodic and semantic qualities: They occurred at a specific time and place, and participants would be expected to remember specific details associated with the event or with their experience learning about it, while at the same time being public events that can be integrated into the knowledge base (cf. Brown, 1990). The forgetting function in episodic memory is well established (Ebbinghaus, 1885); semantic memory, conversely, tends to show much slower loss of information, with some researchers (e.g., Bahrick and colleagues, 2013) arguing for a “permastore,” in which information is stored for very long periods with minimal forgetting. The fact that overall memory for these items did not decline with a two-year delay (albeit with different participants), with the exception of younger adults in the short-answer task, suggests that they are somewhat crystallized in nature. Based on the data reported here and in Coane et al. (2022), where high rates of *remember* and *know* responses were given by participants, memories for public news events appear to exist, at least in part, as a form of general knowledge, but may retain some episodic qualities as well.

Older adults’ memory performance is relevant here as well. The stimuli could be considered somewhat episodic in nature, so finding that older adults can appropriately distinguish between DR and DK is promising, given previous evidence of decreased calibration in episodic FOK in aging (e.g., Morson et al., 2015). As discussed above, the stimuli used here differ in many ways from those used in traditional episodic tasks: not only were they more complex, referring to events rather than single words or word pairs, but the acquisition or encoding phase was likely richer, more meaningful, and distributed. All these factors combined might have created a strong enough memory trace to enable older adults not only to remember the events, but to accurately assess their own memory. It is also possible that the partial semanticization of the public events enabled older adults to rely on their rich knowledge networks for storage and retrieval, which older adults are known to do more so than younger adults (see Umanath & Marsh, 2014; Umanath, 2016), and that this also supported their metacognitive performance. As noted in Introduction, age differences are usually absent in semantic metacognitive tasks. This might provide additional indirect evidence that these public events are, in fact, partially semanticized, although clearly more work needs to be done in this area. To our knowledge, the process of semanticization of episodically acquired information has been primarily examined in younger adults (e.g., Conway et al., 1997; Herbert & Burt, 2004).

### Future directions

Based on the present findings, there are several avenues for future work. One path is to continue to expand the generalizability of the present findings. In fact, the simplicity and intuitive nature of these terms make them potentially well suited for examining the phenomenology of retrieval failures in certain populations, for whom maintaining and processing numerical scales, such as FOKs, might be challenging (e.g., children, adults with cognitive decline or impairment).

Future research should continue to directly examine the utility of this measure. Even though participants define DR and DK the same basic way without context, in the context of crystallized general knowledge (Coane & Umanath, 2019), and in the context of information that likely preserves episodic details while sharing features with semantic memory (the present data), it is still critical to establish what these terms mean to participants in their natural language use in other contexts. For example, in traditional episodic/event memory tasks, such as list-learning paradigms followed by recall or recognition tasks, DK could shift to reflecting something like “I don’t know if I studied that” rather than a lack of availability. Without face validity and consensus between participants and researchers on what DR and DK mean, any results would be indecipherable.

## Conclusions

Following from the Bahrick et al. (2011) model, the present work provides further support of the basic validation of DR and DK as metacognitive tools for capturing the concepts of accessibility- versus availability-based retrieval failures. Moreover, our observation of the nature of participants’ phenomenological reports of their usage of DR and DK solidifies the supposition that participants’ natural language use is fundamentally aligned with these metacognitive concepts once again. Unlike many other metacognitive tools, it seems that definitional instructions are not necessary. However, it is certainly recommended that post-task participant-generated definitions are collected and examined. This practice is consistent with other studies such as prospective memory research practices of ensuring participants are able to identify what the prospective memory task was at the end of the study and removing those who do not (e.g., Einstein et al., 2003; Kvavilashvili et al., 2009; McDaniel et al., 2011), and improvements for the RK paradigm (e.g., McCabe et al., 2011; Migo et al., 2012; Rotello et al., 2005; Umanath & Coane, 2020). Additionally, the present work achieved the goal of behaviorally testing and ultimately validating a new aspect of the generalizability of using DR and DK: Distinguishing between accessibility and availability failures for naturalistically acquired, real-world information that is not squarely semantic knowledge as traditionally understood. Further research remains, and will always remain, to be done to continue to extend the boundaries of the usefulness of these terms for capturing retrieval failures.

## Supplementary Information

**Additional file 1.** Reaction Time of Performance on Initial GK Test: Short-Answer Questions for Waves 1 and 2.

## Data Availability

The dataset(s) supporting the conclusions of this article and the stimuli are available on the Coane Memory and Language Lab’s website: https://web.colby.edu/memoryandlanguagelab/publications/stimuli-and-data-sets/.

## Footnotes

1 Degrees of freedom are corrected for a violation of the equality of variances assumption.
