# Supplementary material for: Ecological validity of don’t remember and don’t know for distinguishing accessibility- versus availability-based retrieval failures in older and younger adults: knowledge for news events
Source: Cogn Res Princ Implic. 2023 Jan 5;8:2. doi: 10.1186/s41235-022-00458-7 (PMC9813323; doi:10.1186/s41235-022-00458-7)
Supplement: Supplementary file 1 — Additional file 1. Reaction Time of Performance on Initial GK Test: Short-Answer Questions for Waves 1 and 2. [file 41235_2022_458_MOESM1_ESM.pdf]

Supplementary Materials

Reaction Time of Performance on Initial GK Test: Short-Answer Questions for Waves 1 and 2

As for the primary analyses, we treated Wave as a between subjects factor. We examined response latencies for the familiarity rating task, the short-answer task, and the final multiple-choice task as a function of age and the response given during the open-ended question task. Mean zRTs (Faust et al., 1999) were analyzed in Age x Response x Wave mixed ANOVAs.

Familiarity. The analyses included data from 26 YAs and 32 OAs in Wave 1 and 37 YAs and 38 OAs in Wave 2. Average latencies varied as a function of subsequent response, $F(2.14, 276.18) = 3.29$, $p = .035$, $\eta_p^2 = .02$. In general, correct responses and DK responses were faster than DR and Incorrect responses, although none of the pairwise comparisons were significant, all ps ≥ .073. The only other significant effect was the age by response interaction, $F(3, 387) = 7.32$, $p = .001$, $\eta_p^2 = .05$. As can be seen in Table S1, the interaction reflects the fact that younger adults responded more quickly when the item was subsequently correctly recalled or not remembered, but were slightly (albeit not significantly) slower when it was not known and significantly slower in subsequent incorrect responses ($p = .004$ and $p = .016$), $F(3, 127) = 5.78$, $p < .001$, $\eta_p^2 = .12$, whereas older adults took significantly longer to rate the familiarity of not remembered items relative to both correctly recalled ($p = .037$) and to not known items ($p < .001$) , $F(3, 127) = 6.93$, $p < .001$, $\eta_p^2 = .14$. None of the other pairwise comparisons were significant, ps ≥ .409. Thus, whereas younger adults took similar amounts of time to determine the familiarity of information they did not know, did not remember, or correctly recalled but were significantly slower at determining the familiarity of information they answered

incorrectly in the subsequent task, older adults seemed to rate the familiarity of correct, incorrect, and not known information at similar speeds and took more time to assess not remembered information, indicating they were engaging in a search for the information. No other main effects or interactions were significant, all *F*s ≤ 1.89, *p*s ≥ .130.

Table S1.

Mean Standardized Response Latencies in the Familiarity Task as a Function of Age and Response Given during the Short-Answer Task (Standard Errors in Parentheses) in Waves 1 and 2

		Wave 1	Wave 2	Average
Younger Adults				
	Correct	-.06 (.04)	-.05 (.04)	-.06 (.03)
	DR	-.09 (.05)	-.01 (.04)	-.05 (.03)
	DK	.08 (.04)	-.02 (.03)	.03 (.02)
	Incorrect	.19 (.09)	.15 (.08)	.17 (.06)
Older Adults				
	Correct	-.06 (.04)	.02 (.04)	-.01 (.03)
	DR	.08 (.05)	.12 (.04)	.10 (.03)
	DK	-.06 (.04)	-.09 (.03)	-.08 (.02)
	Incorrect	.05 (.008)	-.09 (.08)	-.02 (.06)

Short-Answer Task. The analyses yielded a significant effect of response, $F(2.36, 304.16) = 160.39$, $p < .001$, $\eta_p^2 = .55$: DK responses (*M* = -.48, *SEM* = .01) were fastest, followed by DR responses (*M* = .12, *SEM* = .04), then by correct responses (*M* = .39, *SEM* = .04), and finally by incorrect responses (*M* = .63, *SEM* = .05). All pairwise comparisons were significant (all *p*s < .001; see Table S2). Thus, assessments of *not knowing* were made quickly, whereas *not remembering* or retrieval of an answer, regardless of accuracy, took more time. Incorrect responses, in fact, were the slowest, even though, as indicated by the familiarity rating task, they were rated as equally familiar as items not remembered. Thus, response latencies indicate that retrieval of an incorrect answer is not only slower than retrieval of correct answers, but

also than assessments of failures in access. Even though response times were standardized, older adults (*M* = .11, *SEM* = .03) were significantly faster than younger adults (*M* = .22, *SEM* = .03) overall, $F(1, 129) = 7.25$, $p = .008$, $\eta_p^2 = .05$. This main effect was qualified by a Wave by Age interaction, $F(1, 129) = 6.02$, $p = .016$, $\eta_p^2 = .04$. Older adults' response times did not differ as a function of Wave, F = 1.32, p = .25, whereas younger adults were slower in Wave 2 than in Wave 1, $F(1, 129) = 5.22$, $p = .024$, $\eta_p^2 = .04$.

Table S2.

Mean Standardized Response Latencies as a Function of Age and Response in the Short-Answer Task (Standard Errors in Parentheses) in Waves 1 and 2

		Wave 1	Wave 2	Average
Younger Adults				
	Correct	.33 (.08)	.54 (.08)	.39 (.06)
	DR	.23 (.08)	.15 (.07)	.19 (.06)
	DK	-.46 (.03)	-.44 (.02)	-.45 (.02)
	Incorrect	.59 (.11)	.92 (.10)	.76 (.08)
Older Adults				
	Correct	.43 (.09)	.33 (.08)	.38 (.06)
	DR	.11 (.08)	.01 (.07)	.06 (.05)
	DK	-.49 (.02)	-.55 (.02)	-.52 (.02)
	Incorrect	.51 (.11)	.50 (.10)	.51 (.07)

Multiple Choice Task. The analyses on response times in the final multiple-choice test included 24 YAs and 32 OAs in Wave 1 and 33 YAs and 38 OAs in Wave 2. Response latencies varied as a function of initial response, $F(1.87, 229.46) = 98.24$, $p < .001$, $\eta_p^2 = .44$ (see Figure S1). Initially correct responses (*M* = -.58, *SEM* = .02) were significantly faster than initial DR responses (*M* = -.03, *SEM* = .03, $p < .001$), which in turn were faster than initial DK responses (*M* = .20, *SEM* = .03, $p < .001$). Initial Incorrect responses (*M* = .28, *SEM* = .07) were slower than initial correct and DR responses (both *p*s < .001) but similar to initial DK responses ($p \geq .999$).

Older adults (M = -.09, SEM = .03) were faster overall than younger adults (M = .03, SEM = .03), $F(1, 123) = 7.44$, $p = .007$, $\eta_p^2 = .06$. None of the other main effects or interactions were significant, all *F*s ≤ 1.68, all *p*s ≥ .17. Overall, both age groups correctly answered items they had previously correctly retrieved rapidly and were slower at correctly answering items that had been deemed inaccessible, with the slowest correct responses occurring for items that were not known or resulted in errors, indicating the likely use of sophisticated guessing or exclusion strategies. These results suggest that DR does reflect a failure in accessibility, because, once the answer is provided in a multiple-choice test, it is recognized more quickly than an item identified as not known.

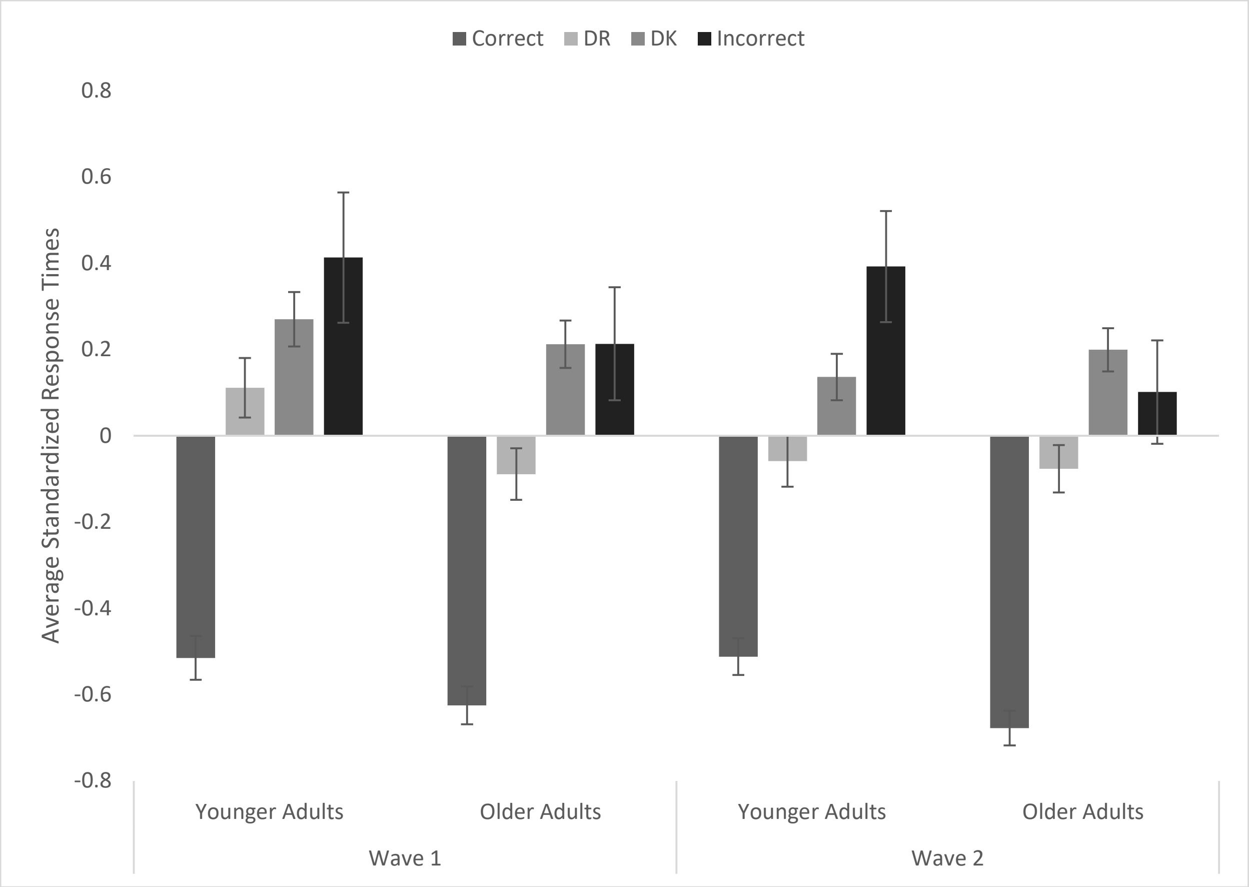

Figure S1. Z-scored mean response times to correct responses on the final MC test in Waves 1 and 2 as function of initial test response and age (error bars reflect standard error of the mean).
